# Preparation of LiFePO_4_/C Cathode Materials via a Green Synthesis Route for Lithium-Ion Battery Applications

**DOI:** 10.3390/ma11112251

**Published:** 2018-11-12

**Authors:** Rongyue Liu, Jianjun Chen, Zhiwen Li, Qing Ding, Xiaoshuai An, Yi Pan, Zhu Zheng, Minwei Yang, Dongju Fu

**Affiliations:** 1 Research Institute of Tsinghua University in Shenzhen, High-Tech Industry Park, Nanshan District, Shenzhen 518057, China; zwlihit@163.com (Z.L.); youyou.orange23@163.com (D.F.); 2 Shenzhen Institute of THz Technology and Innovation, Xixiang, Bao’an District, Shenzhen 518102, China; dingqing@huaxunchina.cn (Q.D.); panyi@huaxunchina.cn (Y.P.); zzealot99@gmail.com (Z.Z.); yangpound@163.com (M.Y.); 3 Shenzhen Graduate School, Harbin Institute of Technology, Shenzhen University Town, Xili, Shenzhen 518055, China; anxiaoshuai@126.com

**Keywords:** LiFePO_4_/C composite, cathode material, green synthesis route, lithium-ion batteries

## Abstract

In this work, LiFePO_4_/C composite were synthesized via a green route by using Iron (III) oxide (Fe_2_O_3_) nanoparticles, Lithium carbonate (Li_2_CO_3_), glucose powder and phosphoric acid (H_3_PO_4_) solution as raw materials. The reaction principles for the synthesis of LiFePO_4_/C composite were analyzed, suggesting that almost no wastewater and air polluted gases are discharged into the environment. The morphological, structural and compositional properties of the LiFePO_4_/C composite were characterized by X-ray diffraction (XRD), scanning electron microscope (SEM), transmission electron microscopy (TEM), Raman and X-ray photoelectron spectroscopy (XPS) spectra coupled with thermogravimetry/Differential scanning calorimetry (TG/DSC) thermal analysis in detail. Lithium-ion batteries using such LiFePO_4_/C composite as cathode materials, where the loading level is 2.2 mg/cm^2^, exhibited excellent electrochemical performances, with a discharge capability of 161 mA h/g at 0.1 C, 119 mA h/g at 10 C and 93 mA h/g at 20 C, and a cycling stability with 98.0% capacity retention at 1 C after 100 cycles and 95.1% at 5 C after 200 cycles. These results provide a valuable approach to reduce the manufacturing costs of LiFePO_4_/C cathode materials due to the reduced process for the polluted exhaust purification and wastewater treatment.

## Highlights:

✔ LiFePO_4_/C is synthesized by using a green route where almost no wastewater and air polluted gases are discharged into the environment.
✔ The reaction principles for the synthesis of LiFePO_4_/C are analyzed.
✔ LiFePO_4_/C exhibits uniform nano-structure and carbon layer.
✔ LiFePO_4_/C shows excellent rate capability and cycling capability.

## 1. Introduction

Olivine-type LiFePO_4_ is considered as one of the most promising cathode materials for Li ions batteries owing to its high operating voltage (~3.4 V vs. Li/Li^+^), high theoretical capacity (~170 mA h/g), low cost and no environmental pollution [1,2,3,4,5,6,7]. However, bare LiFePO_4_ materials suffer from many disadvantages, such as low conductivity and sluggish diffusion rate of Li^+^ ions coupled with low tap density [6,7]. Recently, many efforts have been made to improve its conductivity and accelerate the diffusion rate of Li^+^, including coating the conducting materials on the surface of LiFePO_4_ materials [8,9,10,11], reducing particle size [12], doping transition metals ions [13], etc. Ultimately, high-quality LiFePO_4_ materials have been successfully developed and commercialized in energy storage and electric vehicles (EVs).

However, there still exist some challenging problems for the commercialization of LiFePO_4_ materials in the next generation of lithium-ion batteries. Firstly, complex fabrication procedures such as ingredients, pulping, coating, tableting, winding and assembly welding, further need to be simplified and optimized [7,14]. Secondly, understanding the kinetic behavior of LiFePO_4_ material for the lithium-ion batteries is of fundamental importance [2,6], including the conductive pathway with conducting materials coated on its surface, the Li^+^ ions diffusion dynamics with the transition metal atoms doping, the Li^+^ ions diffusion pathway during the insertion/extraction process, etc. Thirdly, reducing the manufacturing costs of LiFePO_4_ materials and preventing environmental pollution are quite important. Currently, it is noted that the synthesis LiFePO_4_ materials always uses solid state reaction method [15,16], liquid phase method [17], sol-gel method [18], hydrothermal method [19,20] and spray pyrolysis method [21,22]. Almost all these methods can produce wastewater containing excessive anions impurities such as SO_4_^2−^, Cl^−^ and NO_3_^−^, and contaminated gas (N_x_O_y_, CO, and NH_3_), which need additional apparatus to deal with them and increase the manufacturing cost. Therefore, seeking approaches to further reduce the manufacturing cost of LiFePO_4_ materials synthesis and preventing environmental pollution are still highly pursued by materials scientists.

Here, we developed a green route to synthesize the LiFePO_4_/C composite by using Iron (III) oxide (Fe_2_O_3_) nanoparticles, Lithium carbonate (Li_2_CO_3_), glucose powder and phosphoric acid (H_3_PO_4_) solution as raw materials. We first synthesized the FePO_4_·2H_2_O precursor by the reaction of Fe_2_O_3_ nanoparticles with H_3_PO_4_ solution. The wastewater was water and excessive H_3_PO_4_ solution which could be recycled next time. Second, we synthesized the LiFePO_4_/C composite by annealing the mixtures composed of FePO_4_·2H_2_O precursor, Li_2_CO_3_ and glucose powder at a high-temperature process, where only CO_2_ gas and water vapor were discharged. Therefore, all the reaction processes were environmentally friendly. The morphological, structural, compositional properties of the synthesized LiFePO_4_/C composite were characterized. Lithium-ion batteries using such composite as cathode active materials were fabricated, and the corresponding electrochemical performance were discussed.

## 2. Experimental Section

### 2.1. Preparation of LiFePO_4_/C Composite

Iron (III) oxide (Fe_2_O_3_) powder (~800 nm (ACS, 99.99%)), phosphoric acid (H_3_PO_4_) solution (85%), Lithium carbonate (Li_2_CO_3_) and glucose powder were purchased from Sigma Aldrich (Shanghai, China) and used without further purification unless stated otherwise. The FePO_4_·2H_2_O precursor was prepared by the chemical reaction of Fe_2_O_3_ powder with H_3_PO_4_ solution where the molar ratio of the Fe/P was 1:1.05. In a typical procedure, 16 g of Fe_2_O_3_ powder and 14.4 mL H_3_PO_4_ solution were added into 20 mL deionized water in a flask followed by ultrasonic dispersion for 30 min. Then, the mixed slurries were transferred into a ball mill tank and ball-milled for additional 9 h. After that, the mixed slurries were filtered and allowed to heat up to 85 °C for 5 h forming a suspension, followed by cooling down to room temperature. The white precipitate (FePO_4_·2H_2_O precursor) was collected and separated by centrifugation and washed with water for several times, and then dried in a blast drying box for 24 h. The LiFePO_4_/C composite were prepared by using stoichiometric amounts of FePO_4_·2H_2_O precursor, Li_2_CO_3_ and glucose powder (60.0 g glucose/1 mol FePO_4_·2H_2_O precursor) as the starting materials, followed by high temperature sintering. First, stoichiometric amounts of FePO_4_·2H_2_O precursor and Li_2_CO_3_ were added into water-dissolved glucose solution in a flask followed by ultrasonic dispersion for 30 min. Then, the mixed slurries were dried in a blast drying box for 24 h. After that, the mixture was sintered at 650 °C in a tube furnace for 10 h under argon flow to obtain LiFePO_4_/carbon composite.

### 2.2. Characterization of LiFePO_4_/C Composite

The crystallinity was estimated by using X-ray diffraction (XRD, D/Max-IIIC, Rigaku Co., Tokyo, Japan) equipped with a Cu-Kα source of wavelength λ = 1.54060 Å and operated at 40 kV and 20 mA. The top-view SEM images were taken on a Hitachi S-4800 (Hitachi Limited, Tokyo, Japan), and the attached energy dispersive spectrometer (EDS (Hitachi Limited, Tokyo, Japan) analyzer was used to analyze the composition distribution of carbon. The transmission electron microscopy (TEM) images were acquired on a FEI Talos F200X (FEI, Hillsboro, OR, USA) with an acceleration voltage of 200 kV. Thermo-Gravimetric coupled with Differential Scanning Calorimetry (TG-DSC, Netzsch Scientific Instruments Trading Co., Ltd., Shanghai, China) was used to measure the carbon content in the LiFePO_4_/carbon composite. Raman spectra were tested at room temperature equipping with 514 nm laser excitations. X-ray Photoelectron Spectroscopy (XPS) measurement was performed on a SPECS HSA-3500 (SPECS, Berlin, Germany) to determine the valence state of each element of the samples. 

### 2.3. Electrochemical Measurements

The electrochemical measurements were performed using a CR2032 coin-type cell assembled in an argon-filled glove-box (MIKROUNA, Guangzhou, China). For fabricating the working electrodes, a mixture of active materials (LiFePO_4_/C composite), conductive carbon blacks (Super-P, Shenzhen, China), and polyvinylidene fluoride (PVDF) binder at a weight ratio of 80:10:10 was coated on aluminum foil and dried in vacuum at 120 °C for 12 h. The thickness and loading level were 48 µm and 2.2 mg/cm^2^, respectively. The lithium pellets were used as the counter and reference electrode. The electrolyte consisted of a solution of 1 mol/L LiPF_6_ in ethylene carbon (EC)/dimethyl carbonate (DMC) (1:1 *w*/*w*). A celguard 2300 microporous film was used as separator. The cells were galvanostatically charged and discharged between 2.5 V and 4.2 V versus Li/Li^+^ on a battery cycler (LAND, CT2001A, Wuhan, China). Cyclic voltammogram (CV) measurements were carried out using a Multi Autolab electrochemical workstation (Metrohm, Guangzhou, China) at a scanning rate of 0.1–0.5 mV s^−1^. Electrochemical impedance spectra (EIS) were also characterized by Autolab electrochemical workstation adjusting amplitude signal at 5 mV and frequency range of 0.01 Hz–100 kHz.

## 3. Results and Discussions

**Figure 1:**
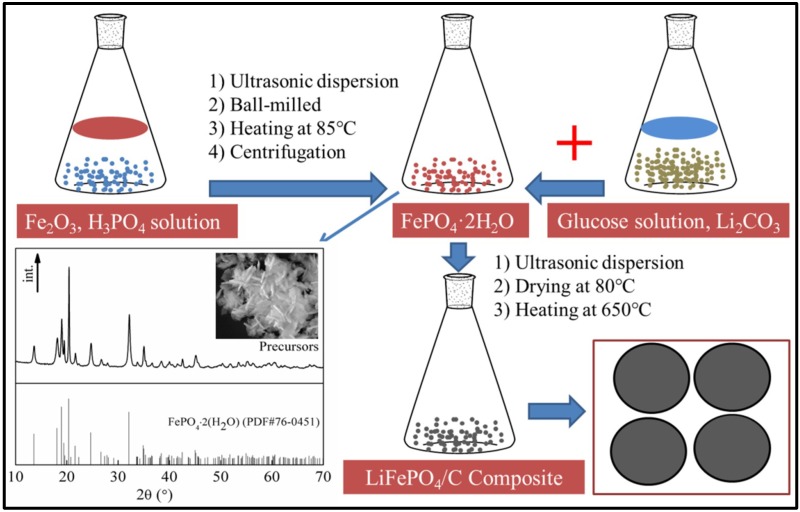
Schematic illustration for the preparation of the LiFePO_4_/C composite.

Figure 1 shows the schematic diagram of the preparation of LiFePO_4_/C composite. Firstly, an excess of phosphoric acid solution reacted with Iron (III) oxide (Fe_2_O_3_) powder to form FePO_4_·2H_2_O precursors. Subsequently, these precursors were mixed with Lithium carbonate (Li_2_CO_3_) and glucose powder followed by high temperature sintering to form LiFePO_4_/C composite. The reaction equations are shown below.

(1) $${\mathrm{Fe}}_{2}O_{3}+2H_{3}{\mathrm{PO}}_{4}+H_{2}O\to {\mathrm{FePO}}_{4}\cdot 2H_{2}O.$$

(2) $$2{\mathrm{FePO}}_{4}\cdot 2H_{2}O+{\mathrm{Li}}_{2}{\mathrm{CO}}_{3}+C_{6}H_{12}O_{6}\to 2{\mathrm{LiFePO}}_{4}/C+\text{volatile matter}$$

Equation (1) shows the synthesis process of FePO_4_·2H_2_O precursors. The reaction mechanism is referred to the reported literature [23], where the Fe salts are used and the reaction equations are shown below:

(3) $${\mathrm{Fe}}^{3+}+H_{2}O=\mathrm{Fe}{\left(\mathrm{OH}\right)}^{2+}+H^{+}$$

(4) $$\mathrm{Fe}{\left(\mathrm{OH}\right)}^{2+}+H_{2}O={\mathrm{Fe}{\left(\mathrm{OH}\right)}_{2}}^{+}+H^{+}$$

(5) $${\mathrm{Fe}}^{3+}+H_{3}{\mathrm{PO}}_{4}={\mathrm{FeH}}_{2}{{\mathrm{PO}}_{4}}^{2+}+H^{+}$$

(6) $${\mathrm{FeH}}_{2}{{\mathrm{PO}}_{4}}^{2+}+H_{3}{\mathrm{PO}}_{4}={\mathrm{Fe}{\left(H_{2}{\mathrm{PO}}_{4}\right)}_{2}}^{+}+H^{+}$$

(7) $$\mathrm{Fe}{\left(\mathrm{OH}\right)}^{+}+{\mathrm{Fe}{\left(H_{2}{\mathrm{PO}}_{4}\right)}_{2}}^{+}=2{\mathrm{FePO}}_{4}+2H_{2}O+2H^{+}$$

In this work, we replaced the Fe salts with the Fe_2_O_3_ powder as raw materials to synthesize the FePO_4_·2H_2_O precursors because the wastewater from the above method contains impurity ions such as SO_4_^2−^, Cl^−^ and NO_3_^−^ which are not environmentally friendly, although Fe salts are very cheap. Due to the complex reaction processes, we add Equations (3)–(7) to get Equation (1) where the Fe^3+^ ions are replaced by Fe_2_O_3_. In addition, it takes a little time to dissolve the Fe_2_O_3_ powder in the acid solution, leading to a lower reaction rate of our method in comparison with that of the method described above. In the environmental protection perspective, our method is favorable to the commercialization of future products. The reason is that there are no metal ion and anion impurities left in the wastewater solution. Although the phosphoric acid solution is excessive, it can be recovered and recycled. Equation (2) displays the synthesis process of LiFePO_4_/C composite. The reaction equation is balanced according to the stoichiometric values of Li, Fe and P elements, where LiFePO_4_/C is the final product while the volatile matter represents the volatile gases, such as CO_2_ gas and H_2_O vapor, even a small amount of CO gas, other C_x_H_y_O_z_, etc. To determine whether the CO gas or C_x_H_y_O_z_ is present in the exhaust during the synthesis of LiFePO_4_/C composite, thermodynamic Gibbs free energies for the formation of CO and C_x_H_y_O_z_ were calculated. Because the precursors were annealed in inert gas, the thermal decomposition products of the FePO_4_·2H_2_O are FePO_4_ and H_2_O vapor, while those of Li_2_CO_3_ are Li_2_O and CO_2_, and those of C_6_H_12_O_6_ are C and H_2_O vapor. Therefore, the formation of CO or C_x_H_y_O_z_ can be derived from the reaction of C with H_2_O vapor or CO_2_ gas. By thermodynamic Gibbs free energies calculation, the temperature for the formation of CO gas must be more than 980.6 K. In our experiment, the sintering temperature for the precursors was 650 °C (923 K), i.e. lower than 980.6 K. Thus, CO gas was not generated. C_x_H_y_O_z_ was also not generated due to absence of CO and H_2_. In other words, our work is a green route to synthesize the LiFePO_4_/C cathode materials for lithium ion battery application.

**Figure 2:**
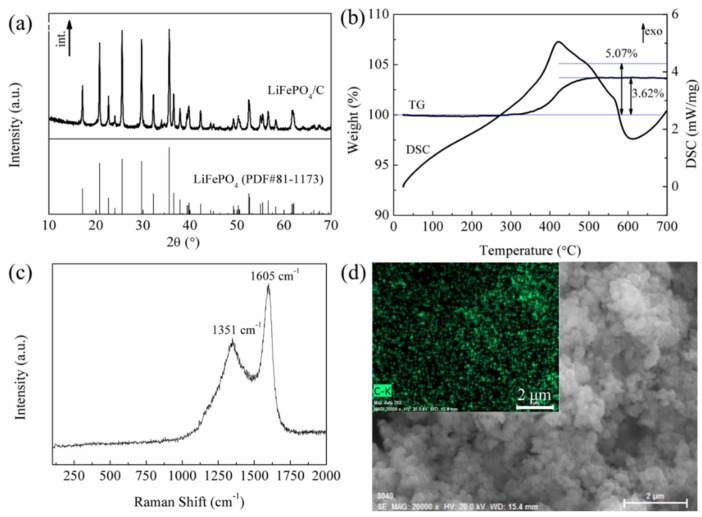
(**a**) XRD pattern of LiFePO_4_/C composite; (**b**) TG-DSC curves of the LiFePO_4_/C composite recorded from the room temperature to 700 °C at a heating rate of 10 °C min^−1^ in air; (**c**) Raman spectrum of LiFePO_4_/C composite; and (**d**) EDS mapping of C in the LiFePO_4_/C composite.

Figure 2a shows the XRD pattern of LiFePO_4_/C composite. As can be seen, all XRD peaks match well with the standard data JCPDS (Joint Committee on Powder Diffraction Standards) card No. 81-1173, demonstrating the formation of LiFePO_4_ with orthorhombic structure. The lattice parameters are a = 10.342 Å, b = 6.021 Å, and c = 4.699 Å, respectively. The main XRD peaks are strong and sharp, suggesting good crystallinity of LiFePO_4_/C composite. The XRD peaks assigned to the carbon are not detected due to its amorphous state [22]. Moreover, its low content also plays an important role. Figure 2b shows the TG/DSC curves to estimate the carbon content in the LiFePO_4_/C composite. As can be seen, the weight gain of 3.62% below 550 °C is assigned to the oxidation of LiFePO_4_/carbon to the Li_3_Fe_2_(PO_4_)_3_ and Fe_2_O_3_ [24,25]. Above 550 °C, there is almost no weight change, indicating that the LiFePO_4_/C composite are fully oxidized where the carbon is oxidated to the CO_2_ gas. According to the total weight gain of 5.07% for pure LiFePO_4_ in theory [26], the amount of carbon in the LiFePO_4_/C composite is about 1.45%. Figure 2c shows the Raman characterization of LiFePO_4_/C composite. The Raman spectrum exhibits two peaks at 1351 cm^−1^ and 1605 cm^−1^ corresponding to the D band (disordered carbon, sp3) and G band (graphite, sp2) for amorphous carbon, respectively [27,28,29,30]. The observed D band and G band indicate the existence of carbon in the LiFePO_4_/C composite. A lower relative intensity ratio of D/G band corresponds to a higher order carbon arrangement. As can be seen, the relative intensity ratio of D/G is 0.66 and the G band shows a smaller full-width half-maximum compared to that of the D band, indicating high graphitization of C in the LiFePO_4_/C composite. Although the Raman spectrum shows a sharp graphitic carbon peak, the carbon remains in the amorphous state in the LiFePO_4_/C composite. Therefore, it is not detected by XRD characterization. Figure 2d shows EDS mapping to estimate the composition distribution of carbon element in the LiFePO_4_/C composite. As can be seen, the carbon is uniformly distributed across the whole surface, which is beneficial to the conductivity properties of LiFePO_4_ and improves electrochemical performance of Lithium-ion battery.

**Figure 3:**
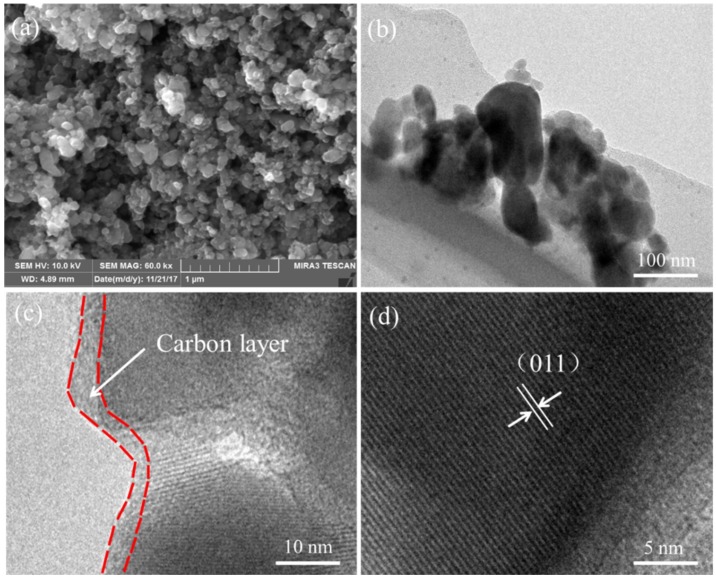
(**a**) SEM image of LiFePO_4_/C composite; and (**b**–**d**) TEM images of LiFePO_4_/C composite.

Figure 3a shows the SEM images of LiFePO_4_/C composite. As can be seen, the LiFePO_4_/C composite exhibit uniform particle size distribution ranging from 100 to 200 nm. The small grain sizes of LiFePO_4_/C composite are attributed to the carbon coating on the surface of the LiFePO_4_ nanoparticles that prevents their quick growth. This phenomenon can be explained by the space steric effect which increases the diffusion activation energy of the reactants and slows down the growth rate of grains [31]. Therefore, the carbon coating layer is quite important in controlling particle size. The small grain sizes are conducive to shortening the migration paths of lithium ions and electrons during the lithiation/delithiation process and as a result, improve the electrochemical performances of LiFePO_4_/C composite efficiently [32]. Further characterization was carried out by TEM and the corresponding images of the LiFePO_4_/C composite are shown in Figure 3b–d. The carbon layer on the LiFePO_4_ nanoparticles surface is uniform, showing a thickness of about 2–3 nm, which demonstrates that the carbon exists in the LiFePO_4_/C composite. This result is consistent with the previous TG-DSC analysis and Raman characterization. The effect of the carbon layer is beneficial to smoothing electron migration for the reverse reaction of Fe^3+^ to Fe^2+^. In addition, the carbon layer can supply a better electronic contact between the LiFePO_4_ nanoparticles, which ensures that the electrons are able to migrate quickly enough from all sides [32,33,34]. Meanwhile, the lattice fringes corresponding to the (011) crystal plane demonstrate the formation of olivine-type LiFePO_4_. 

**Figure 4:**
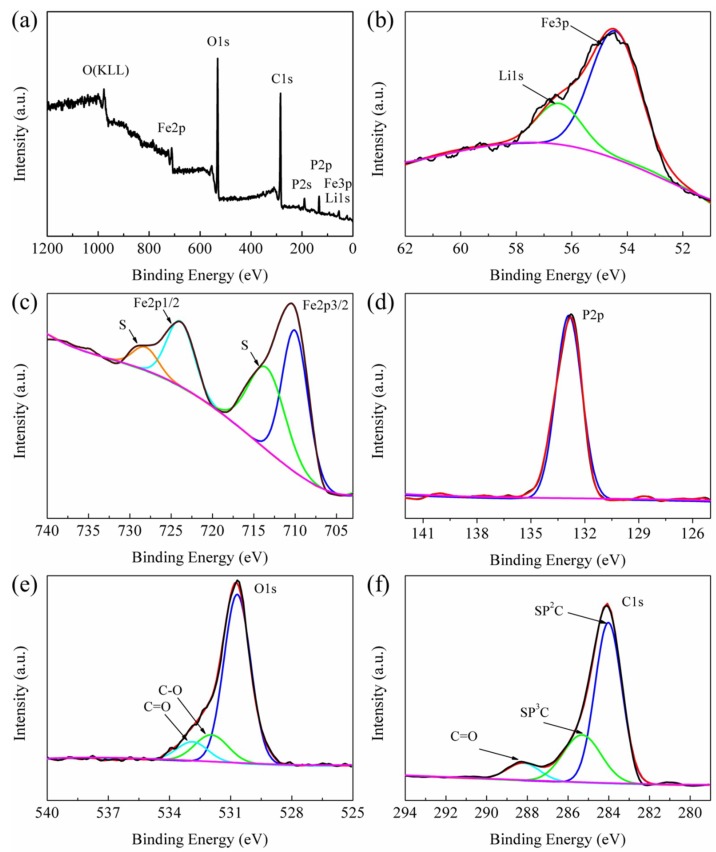
XPS survey of LiFePO_4_/C composite (**a**); high resolution XPS spectrum of: Li 1s (**b**); Fe 2p (**c**); P 2p (**d**); O 1s (**e**); and C 1s (**f**) for LiFePO_4_/C composite.

Figure 4 shows the high-resolution X-ray photoelectron spectroscopy (XPS) spectra of the Li 1s, Fe 3p, Fe 2p, P 2p, O 1s and C 1s core levels to determine the oxidation states of the elements in the LiFePO_4_/C composite. The peak at 56.5 eV, corresponding to the lithium of the LiFePO_4_/C composite, cannot be seen due to the superposed iron peak of Fe 3p [35,36]. The peak intensity of Fe 3p is higher than Li 1s because the Fe 3p has greater relative atomic sensitivity than that of Li 1 s [37,38]. The Fe 2p shows two peaks at 710.1 (2p3/2) and 724.1 eV (2p1/2) with a splitting energy of 14.0 eV, which is close to the standard splitting energy of 19.9 eV, demonstrating the oxidation state of Fe^2+^ [36,38]. Moreover, two small peaks at high binding energy of 713.9 and 728.5 eV are the characters of transition metal ions with partially filled-d orbits, which are assigned to the multiple splitting of the energy levels of Fe ion [37,38]. The peaks representing the other valence states of Fe ions cannot be seen, revealing that only Fe^2+^ ions exist in the LiFePO_4_/C composite. The P 2p shows a peak at 132.9 eV, revealing that the valence state of P is 5+ [38]. The O 1s shows a peak at 531.0 eV, confirming that the valence state of O in the LiFePO_4_/C composite is divalent. The two shoulder peaks at 531.9 and 533.0 eV are attributed to the C–O and C=O bands arising from functional groups absorbed on the sample surface [39]. The C 1s shows peaks at 284.0 and 284.4 eV, which correspond to the short-order sp2-coordinated and sp3-coordinated carbon atoms [38]. The additional peak at 288.2 eV is the C=O band arising from functional groups absorbed on the sample surface. These results confirm that the LiFePO_4_/C composite was synthesized.

**Figure 5:**
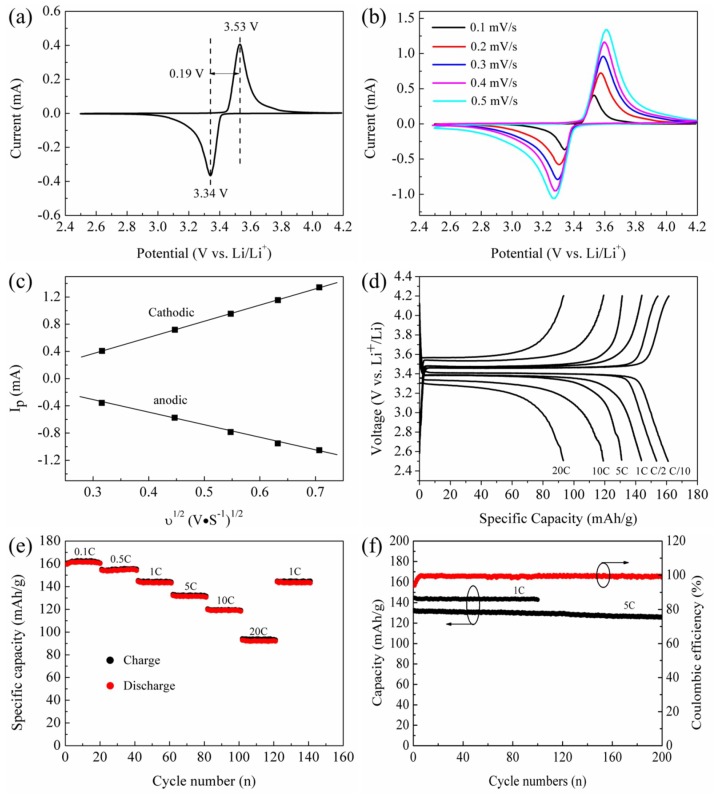
(**a**) Typical CV curve of LiFePO_4_/C composite at scan rate of 0.1 mV/s; (**b**) CV curves of LiFePO_4_/C composite at scan rates of 0.1–0.5 mV/s; (**c**) linear response of the peak current (I_p_) as a function of the square root of scanning rate (ν); (**d**) charge and discharge profiles of LiFePO_4_/C composite in the potential region from 2.5 to 4.2 V at various rates; (**e**) rate performance curves from 0.1 C to 20 C; and (**f**) cycling performance combined with coulombic efficiency at 1 C and 5 C.

Figure 5a shows the cyclic voltammetry curves of lithium ion batteries using the LiFePO_4_/C composite as the cathode active materials. N peak appears at 2.63 V (characteristic of Fe^3+^ in Fe_2_O_3_), indicating that all the iron atoms in the LiFePO_4_/C composite are Fe^2+^ [40]. The two peaks around at 3.34 and 3.53 V (vs. Li^+^/Li) are attributed to the Fe^2+^/Fe^3+^ redox reaction, which corresponds to lithium extraction and insertion in LiFePO_4_ crystal structure [41]. Furthermore, the two peaks show a narrow potential separation of 0.19 V and exhibit good symmetric and poignant shape, which imply a good electrochemical performance for lithium ion batteries. Figure 4b further shows the evolution of the cyclic voltammetry curves of LiFePO_4_/C composite in the scanning rate ranging from 0.1 to 0.5 mV·s^−1^. The peak position shifts and the potential separation between two peaks broadens gradually as the scan rate increases. Previous literature has reported that the diffusion coefficient of lithium ions (D_Li_) can be determined from a linear relationship between peak currents (i_p_) and the square root of the scan rate (v^1/2^) based on the Randles–Sevcik equation [41,42,43]:

(8) $$I_{p}=2.69\times 10^{5}n^{3/2}{\mathrm{ACD}}^{1/2}v^{1/2}$$

where I_p_ (A) is the current maximum, n is the number of electrons transfer per mole (n = 1), F (C/mol) is the Faraday constant, A (cm^2^) is the electrode area (1.77 cm^2^), C (mol/cm^3^) is the lithium concentration in the LiFePO_4_/C composite, v (V/s) is the scanning rate, D_Li_ (cm^2^/s) is the lithium diffusion coefficient, R (J/K·mol) is the gas constant, and T (K) is the temperature. Figure 4c shows the linear relationship between peak currents (I_p_) and the square root of the scan rate (v^1/2^). The diffusion coefficient D_Li_ are calculated to be 4.35 × 10^−13^ and 2.57 × 10^−13^ cm^2^/s for the charge and discharge processes, respectively, which are comparable to the previous reported literature [43,44,45]. This confirms that Li ions show excellent transmission performance, suggesting excellent electrochemical performance of our Li-ion batteries. Figure 4d,e shows the charge/discharge curves of lithium ion batteries at current rate from 0.1 C to 20 C. Apparently, at a low current rate of 0.1 C, the batteries deliver a discharge capacity of 161 mAh·g^−1^, corresponding to 95% of the theoretical capacity (170 mAh·g^−1^) of LiFePO_4_. With the current rate increasing, the discharge capacity continually decreases, which is attributed to the low electronic conductivity and ion diffusion coefficient coupled with low tap density [32,38]. Despite this, the discharge capacity of our lithium ion batteries can reach 119 and 93 mAh·g^−1^ at high current rate of 10 C and 20 C. In addition, our batteries retain an approximate discharge capacity of 161 mAh·g^−1^ at the current rate of 0.1 C after the batteries are tested at the current rate of 20 C. This indicates that our batteries are highly structural stability, which can be suitable for the large current discharge. Figure 4f displays the cyclic performances and the coulombic efficiency of the lithium ion batteries. It is found that the batteries show a discharge capacity of 142 mAh·g^−1^ with a capacity retention of 98% after 100 cycles at 1 C. When the rate reaches at 5 C, the batteries even show discharge capacity of 125 mAh·g^−1^ with a capacity retention of 95.1% after 200 cycles. The coulombic efficiency with a value of 99% almost remains constant. These results demonstrate the high cycling stability of our batteries.

**Figure 6:**
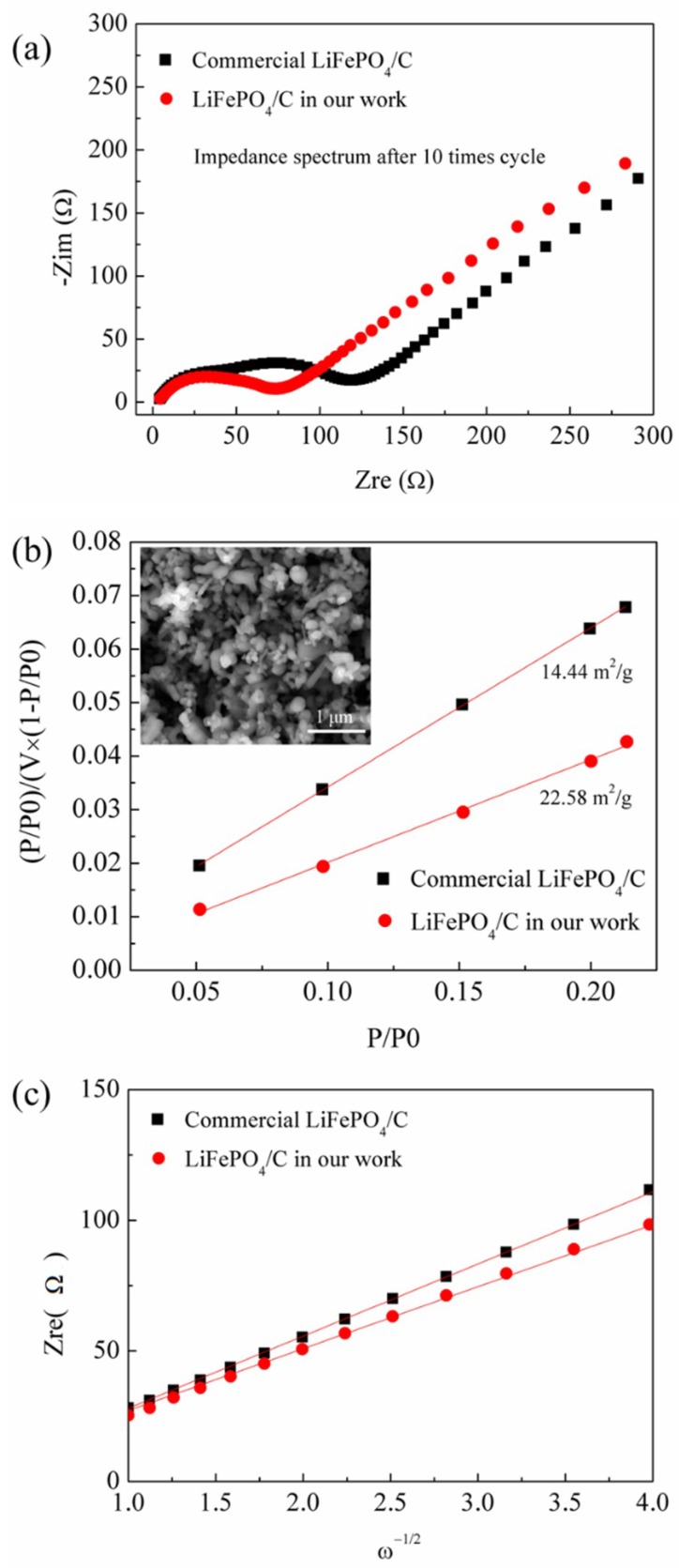
(**a**) The electrochemical impedance spectra (EIS); (**b**) variations and fittings between Z_Re_ and ω^−1/2^ (the reciprocal square root of the angular frequency ω) in the low-frequency region; and (**c**) specific surface area test (insert is the SEM image of commercial LiFePO_4_/C) of our LiFePO_4_/C composite in comparison with those of the commercial LiFePO_4_/C composite.

The electrochemical impedance spectra (EIS) technology is one of the most powerful tools to study electrochemical reactions, such as the processes occurring at the interface between electrodes and electrolyte, and the Li^+^ intercalation/de-intercalation in the interior of cathode/anode materials [46,47]. Figure 6a shows the EIS curve of lithium ion batteries using the LiFePO_4_/C composite as the cathode active materials after 10 cycles at rate of 1 C. Clearly, the EIS curve consists of a semicircle in the high-frequency region followed by a straight line in the low-frequency region. The former is related to the charge-transfer process at the electrode/electrolyte interfaces, while the latter represents the Warburg impedance associated with the Li^+^ diffusion in the LiFePO_4_ crystal lattice [48,49]. The radius of the semicircle in the EIS curve for the LiFePO_4_/C composite is 60.2 Ω. As a comparison, the EIS curve of the commercial LiFePO_4_/C materials is also plotted in Figure 6a. All the procedures for the fabrication of lithium ion batteries are completed under identical conditions. In addition, the loading level of commercial LiFePO_4_/C composite as active materials is also 2.2 g/cm^2^. The commercial LiFePO_4_/C materials with the carbon content of about 1.44% are purchased from the Optimumnano Energy Co., Ltd. (Shenzhen, China). The grain size of the LiFePO_4_/C is 200–300 nm, as shown in Figure 6b. As can be seen, the radius of the semicircle in the EIS curve is 124.2 Ω. This indicates that our LiFePO_4_/C composite shows better electrical properties than that of the commercial LiFePO_4_/C materials. One of the possible reasons is that our LiFePO_4_/C composite (100–200 nm) exhibits relatively smaller grain sizes and higher specific surface area (Figure 6b) in comparison with that of the commercial LiFePO_4_/C materials. This is because the small grain sizes are conducive to shortennig the migration paths of lithium ions and electrons during the lithiation/delithiation process [38]. In addition, the carbon content is very similar between our LiFePO_4_/C composite and the commercial LiFePO_4_/C materials. The diffusion coefficient of Li^+^ (D) can also be calculated form the EIS curve by using the following equation [49,50]:

(9) $$D=R^{2}T^{2}/2A^{2}n^{4}F^{4}C^{2}\sigma^{2}$$

where R is gas constant (8.314 J·mol^−1^·k^−1^), T is the absolute temperature (298.15 K), A is the area of the tested electrode surface (cm^2^), n is the number of electrons involved in the redox process (n = 1 in this work), C is the molar concentration of Li^+^ in the tested electrode, F is the Faraday constant, and σ is the Warburg impedance coefficient [46,47]. By linear fitting the relation plot between Z_Re_ and ω^−1/2^ (the reciprocal square root of the angular frequency ω) (as shown in Figure 6b) to estimate the Warburg impedance coefficient σ, the diffusion coefficient of Li^+^ (D) could be obtained from the above equation. By calculation, the diffusion coefficient of Li^+^ (D) for our LiFePO_4_/C composite is 3.17 × 10^−13^ cm^2^/s. This result is consistent with the previous calculation using the Randles–Sevcik equation. The D value for the commercial LiFePO_4_/C materials is also calculated to be 2.34 × 10^−13^ cm^2^/s. For a comparison, our LiFePO_4_/C composite shows a relatively higher D value, which is assigned to the smaller grain sizes that are conducive to shortening the migration paths of lithium ions [38].

## 4. Conclusions

In conclusion, high-quality LiFePO_4_/C composite were synthesized via a green route in which no wastewater or air polluting gas is discharged into the environment. The synthesized LiFePO_4_/C composite exhibited excellent nanoscale particle size (100–200 nm) showing uniform carbon coating on the surface of LiFePO_4_ nanoparticles, which effectively improved the conductivity and diffusion of Li^+^ ions of LiFePO_4_. Consequently, lithium ion batteries using the as-synthesized LiFePO_4_/C composite as cathode materials exhibit superior electrochemical performance, especially for high rate performance. More importantly, this work provides a valuable method to reduce the manufacturing cost of the LiFePO_4_/C cathode materials due to the reduced process for the polluted exhaust purification and wastewater treatment, which is highly desired for applications such as large-scale energy storage and electric vehicles.

## Prime Novelty Statement

In this work, we develop a green route to synthesize the LiFePO_4_/C composite by using Iron (III) oxide (Fe_2_O_3_) nanoparticles, Lithium carbonate (Li_2_CO_3_), glucose powder and phosphoric acid (H_3_PO_4_) solution as raw materials. In the synthesis process, almost no wastewater and air polluted gases are discharged into the environment and the reaction principles are analyzed. The structural, morphological, compositional properties of the LiFePO_4_/C composite are characterized. Using the LiFePO_4_/C composite as cathode materials for lithium-ion batteries application, excellent electrochemical performances are obtained, showing a discharge capability of 161 mA h/g at 0.1 C, 119 mA h/g at 10 C and 93 mA h/g at 20 C, and a cycling stability with 98.0% capacity retention at 1 C after 100 cycles and 95.1% at 5 C after 200 cycles. These initial research results are very interesting and the technology developed in this work will provide a valuable approach to reduce the manufacturing cost of LiFePO_4_/C cathode materials due to the reduced process for the polluted exhaste purification and wastewater treatment.
